# Insights into phage-bacteria interaction in cold seep *Gigantidas platifrons* through metagenomics and transcriptome analyses

**DOI:** 10.1038/s41598-024-61272-3

**Published:** 2024-05-08

**Authors:** Yan Zhang, Hao Chen, Chao Lian, Lei Cao, Yang Guo, Minxiao Wang, Zhaoshan Zhong, Mengna Li, Huan Zhang, Chaolun Li

**Affiliations:** 1 Center of Deep Sea Research, and CAS Key Laboratory of Marine Ecology and Environmental Sciences, Institute of Oceanology, Chinese Academy of Sciences, Qingdao, 266071 China; 2 National Deep Sea Center, Qingdao, 266071 China; 3 Laboratory for Marine Ecology and Environmental Science, Laoshan Laboratory, Qingdao, 266237 China; 4 South China Sea Institute of Oceanology, Chinese Academy of Sciences, Guangzhou, 510301 China; 5 University of Chinese Academy of Sciences, Beijing, 100049 China

**Keywords:** Viruses, *Gigantidas platifrons*, Metagenome, Transcriptome, Virus–host interaction, Virus-host interactions, Microbiology

## Abstract

Viruses are crucial for regulating deep-sea microbial communities and biogeochemical cycles. However, their roles are still less characterized in deep-sea holobionts. Bathymodioline mussels are endemic species inhabiting cold seeps and harboring endosymbionts in gill epithelial cells for nutrition. This study unveiled a diverse array of viruses in the gill tissues of *Gigantidas platifrons* mussels and analyzed the viral metagenome and transcriptome from the gill tissues of *Gigantidas platifrons* mussels collected from a cold seep in the South Sea. The mussel gills contained various viruses including Baculoviridae, Rountreeviridae, Myoviridae and Siphovirdae, but the active viromes were Myoviridae, Siphoviridae, and Podoviridae belonging to the order Caudovirales. The overall viral community structure showed significant variation among environments with different methane concentrations. Transcriptome analysis indicated high expression of viral structural genes, integrase, and restriction endonuclease genes in a high methane concentration environment, suggesting frequent virus infection and replication. Furthermore, two viruses (GP-phage-contig14 and GP-phage-contig72) interacted with *Gigantidas platifrons* methanotrophic gill symbionts (bathymodiolin mussels host intracellular methanotrophic Gammaproteobacteria in their gills), showing high expression levels, and have huge different expression in different methane concentrations. Additionally, single-stranded DNA viruses may play a potential auxiliary role in the virus–host interaction using indirect bioinformatics methods. Moreover, the Cro and DNA methylase genes had phylogenetic similarity between the virus and *Gigantidas platifrons* methanotrophic gill symbionts. This study also explored a variety of viruses in the gill tissues of *Gigantidas platifrons* and revealed that bacteria interacted with the viruses during the symbiosis with *Gigantidas platifrons*. This study provides fundamental insights into the interplay of microorganisms within *Gigantidas platifrons* mussels in deep sea.

## Introduction

Deep sea represents an extreme and inhospitable environment characterized by darkness, hypoxia, high hydrostatic pressure, and low temperatures, resulting in generally low species^1–3^. However, chemosynthetic environments, including hydrothermal vents and cold seeps, serve as energy hotspots on the seafloor, supporting some of the most unique ecosystems on Earth^4^. These environments are created by the discharge of reduced fluids from the seafloor, often enriched with methane, sulfide, hydrogen, and iron II^5^. These chemicals drive primary production by chemosynthetic microorganisms. Additionally, macrofauna in hydrothermal vents and cold seeps must adapt to the highly toxic chemical environment by acquiring chemosynthetic microbes as symbionts^6^. For example, sponges from the Campos Basin in Southeastern Brazil have endosymbiotic *Nitrosopumilaceae*^7^. Thus far, more than 600 macrofaunal species have been identified in these extremely chemosynthetic environments^8^, with bivalve species, especially mussels, dominating the biomass in these ecosystems^9^. Bathymodiolin mussels harbor endosymbiotic methanotrophs in their gills and derive the vast majority of their nutrition from these symbionts^10,11^. Recent evidence suggests that deep-sea animal holobionts contain a diversity of phages, which could potentially influence animal-bacterial symbioses^12,13^. These intimate symbiotic relationships, common and crucial in vent and seep ecosystems, contribute to the formation of holobionts as adaptations to extreme environments.

Chemosynthetic ecosystems harbor a wide diversity of archaea and bacteria, which play crucial roles in hydrocarbon metabolism^14,15^. These microbial populations drive various biological processes including sulfate reduction, sulfur oxidation, denitrification, metal reduction, and methanogenesis within the seabed^16,17^. Viruses have also been observed in water, sediment, and invertebrates of chemosynthetic ecosystems^18–21^. Investigations from seven cold seep sediment sites have reported a broad range of archaeal and bacterial viruses, such as those for Bathyarchaeota, Methanomicrobia, Thaumarchaeota, Bipolaricaulota, Coatesbacteria, and Sumerlaeota^14^. Additionally, viruses of the Myoviridae, Siphoviridae, Podoviridae, and Microviridae families have been observed in various invertebrates, such as tube worms, sponges, and mussels^13,22–24^. These viruses serve as key agents in natural ecosystems through a range of interactions with their microbial hosts. These interactions manifest in lytic and lysogenic infections of viruses. Lytic infection leads to the reproduction of viral progeny, the lysis of host cells, and the release of cellular dissolved organic matter, thereby influencing microbial community diversity and organic carbon and nutrient turnover^25^. By contrast, temperate viruses replicate alongside their hosts, forming a mutualistic relationship during lysogenic infection^25^. They can reprogram host metabolism by encoding a number of putative auxiliary metabolic genes, affecting processes like sulfur oxidation^26^, phosphate metabolism^27^, pentose phosphate pathway^28^, nitrogen metabolism^29,30^, purine and pyrimidine metabolism pathways^31^, and others. With successive investigations into marine viruses, it has become clear that virus–host interactions are more complex than previously thought, and viruses play essential roles in microbe-driven ocean biogeochemical processes.

Bathymodiolin mussels represent one of the dominant megafaunal taxa found at hydrothermal vents and cold seeps, which provides the microorganisms with optimal growth conditions, necessary metabolites, and shelter^32,33^. They host endosymbiotic bacteria within gill epithelial cells, known as bacteriocytes, and derive energy and nutrients through the oxidation of reducing substances, including methane, hydrogen sulfide, thiosulfate, and hydrogen^34^. Previous studies have primarily focused on the roles of chemoautotrophic bacteria in symbiotic systems, overlooking the presence of viruses. Recent studies, however, have revealed the presence and abundance of viruses, and their interactions with hosts in chemosynthetic ecosystems^14^. Knowledge of the ecological roles of viruses in the deep sea has been constrained by challenges in sampling and extracting viral particles (virions)^35^. In recent years, advancements in sequencing and bioinformatics have enabled the analysis of viruses obtained from metagenomes sequenced without prior virion separation. These methods have significantly advanced viral ecology, from the discovery of novel viruses to understanding their global distribution. In this study, we utilized high-throughput metagenomic and transcriptome sequencing to characterize the viral community composition (only bacterial viruses) and diversity in *Gigantidas platifrons.* We focused on analyzing viral expression, as well as the interactions between viruses and bacteria, to explore the microbial interactions of *G. platifrons* mussels in the deep sea.

## Results and discussion

### Relative abundance and composition of viruses of *G. platifrons*

**Figure 1:**
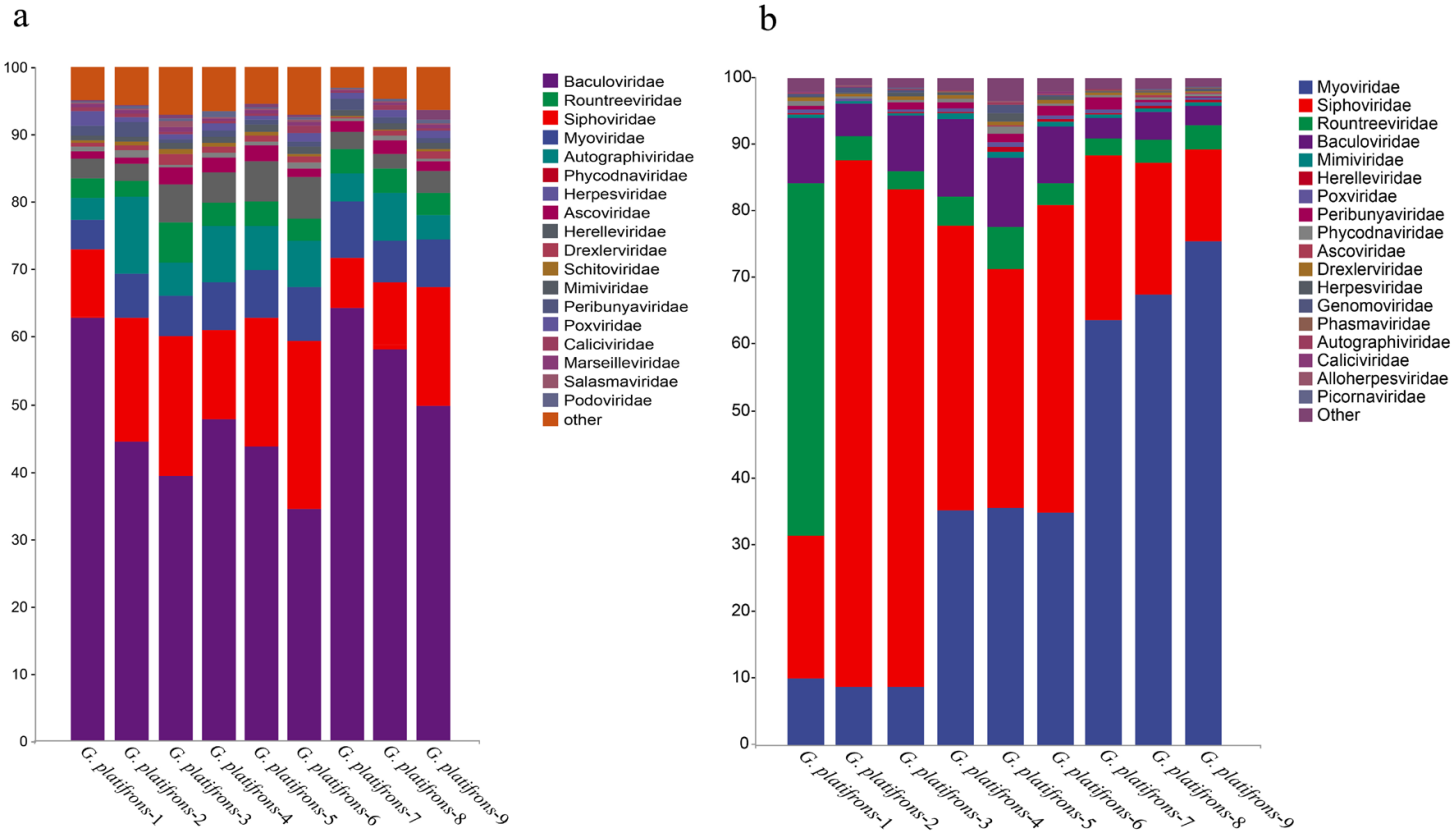
Viral community structure of nine *G. platifrons* individuals at the family level; this analysis was performed on the basis of primary reads (the top 18 reads are shown). (**a**) DNA sequence results; (**b**) RNA sequencing results.

Metagenome and transcriptome data from nine *G. platifrons* samples collected from a cold seep in the South China Sea, in the western Pacific Ocean, were used to identify viromes and active viromes. Kraken 2 identified a total of 86 viral families (Supplementary Table S1). The main viral communities in *G. platifrons* were Baculoviridae, Rountreeviridae, Myoviridae, and Siphovirdae in both DNA and RNA sequencing results, but the active viromes were Myoviridae, Siphovirdae, and Podoviridae belonging to the order Caudovirales (Fig. 1a and b). Although the abundance of viruses varied greatly among different individuals, the species composition of the active viral community remained consistent. This diversity in viral abundance was primarily attributed to samples collected from different environments, suggesting that viral abundance exhibits specificity in various environments and among individuals, while the species composition of the viral community remains relatively uniform (environmental parameters of the samples listed in Table 1). In our study, we specifically discuss the uniformity of the species composition of the activity viral community in *G. platifrons*. Within the Caudovirales order, which includes the families Siphoviridae, Myoviridae, and Podoviridae, Siphoviridae, and Myoviridae were the dominant viral communities in seawater, with variations observed in different seawater areas. Notably, the relative abundance of *Podoviridae* was consistently low in all samples. Conversely, sponge metagenome analysis revealed a higher relative abundance of Podoviridae in the Crane site and Swan site^36^. The second-largest viral communities across all samples belonged to the Rountreeviridae family within the Caudovirales order and the Baculoviridae family within the Lefavirales order, ranging from 1.72 to 21.04% (Supplementary Table S1). The differences in viral communities and diversity in *G. platifrons* may be attributed to variations in deep-sea regions and the bacterial host species within organisms.

**Table 1:** Environmental parameters of the samples.

Individual	Methane (ppm)	DO (mg/L)
*G. platifrons*-1, *G. platifrons*-2, *G. platifrons* -3	Up to 407	3.088
*G. platifrons*-4, *G. platifrons*-5, *G. platifrons*-6	Up to 25,000	3.055–2.910
*G. platifrons*-7, *G. platifrons*-8, *G. platifrons*-9	Up to 4580	3.093

### Viral contigs extracted from the metagenome of *G. platifrons*

**Figure 2:**
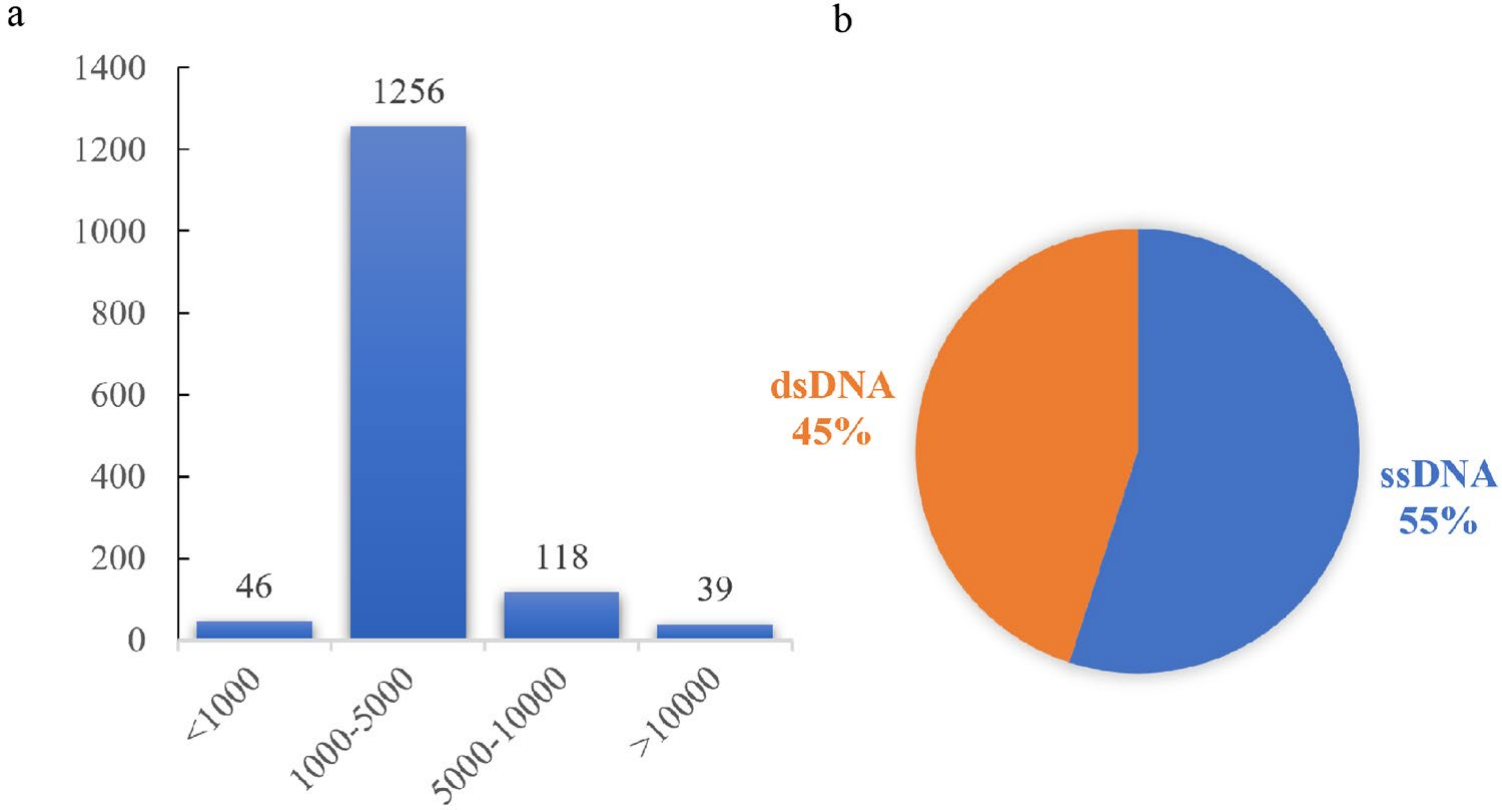
Overview of the viral contigs filtered using the VirSorter2 program. (**a**) The number of viral contigs of different lengths. Abscissa is the length of viral contigs, and ordinate is the number of viral contigs. (**b**) Viral style. The orange color represents dsDNA, and the blue color represents ssDNA.

Metagenomic data were used to assemble reads and identify viral contigs in our study. In total, 1459 contigs were identified from the metagenome using the VirSorter2 program, with a viral hit max_score of ≥ 50 (Fig. 1)^37^. The most contigs range from 1000 to 5000 bp, and 5000–10,000 bp and longer than 10,000 bp are 118 and 39, respectively (Fig. 2a). The longest contig measured 98,882 bp, while the average contig length was 12,905 bp. The viral contigs were categorized into two types of DNA: double-stranded DNA (dsDNA) and single-stranded DNA (ssDNA), constituting 45.23 and 54.76% of the total contigs, respectively (Fig. 2b). Among the 1459 viral contigs, 89 exceeded 5000 bp (Supplementary S2) according to the screening criteria. Additionally, 35 provirus contigs were identified, flanked by host genes on one or both sides (Supplementary Table S2). The viral contigs were classified into two categories: lysis and lysogeny, with lysogenetic viral contigs containing lysogenic genes such as integrase, excision enzyme, and Cro/CI repressor. Our study identified 29 lysogenetic viruses and 60 lysis viruses (Supplementary Table S2).

**Figure 3:**
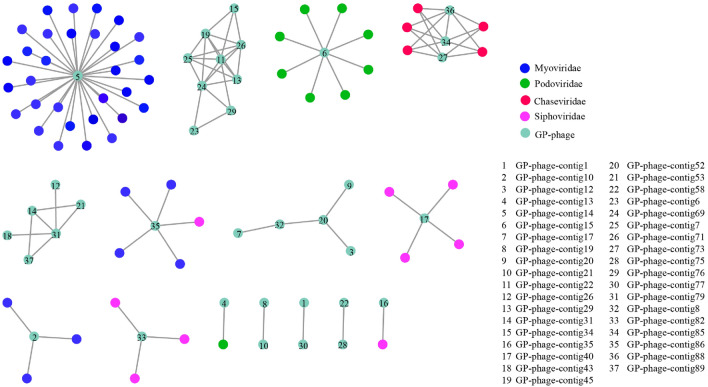
Gene-sharing network of viral contigs constructed using vConTACT2. The figure shows a network of viral contigs studied.

To investigate the community classification of viral contigs in *G. platifrons* from the cold seep, we constructed a gene-sharing network using vConTACT2. A total of 435 viral clusters (VCs) with family-level taxonomy were identified (Supplementary Table S3). Among the 89 viral contigs we studied, 12 clustered with RefSeq virus genomes, implying that these viral contigs can be assumed to belong to the same virus genera as that of the corresponding RefSeq genome. The majority of viral contigs showed no similarity to RefSeq virus genomes, suggesting they may cluster together as novel VCs or as classified as outliers and singletons^38,39^. Compared with the prokaryotic viruses in the RefSeq viral database (V85) (The reference database of vConTACT2 including ProkaryoticViralRefSeq85-ICTV, ProkaryoticViralRefSeq85, ProkaryoticViralRefSeq88, ProkaryoticViralRefSeq94, ProkaryoticViralRefSeq97, ProkaryoticViralRefSeq201, ArchaeaViralRefSeq85, ArchaeaViralRefSeq94, ArchaeaViralRefSeq97, and ArchaeaViralRefSeq201). We selected the viral RefSeq85 as the reference database following Li’s study^14^), the viral contigs were highly novel and variant. The network of viral contigs clustered with Myoviridae (3 sequences), Siphoviridae (4 sequences), Podoviridae (2 sequences), and Chaseviridae (3 sequences; Fig. 3). Myoviridae, Siphoviridae, and Podoviridae were common and prevalent virus populations in the ocean, existing widely in seawater, sediment, and marine invertebrates^40–42^. Myoviridae and Siphoviridae were observed in mussels, clams, tubeworms, snails, and sponges from deep-sea hydrothermal vents and cold seeps^13^. GP-phage-contig14 belongs to Myoviridae and shares similar genes with Enterobacteria phage, which is a virus associated with gut bacteria (Supplementary Table S3). The viral contigs filtered from snail holobionts in hydrothermal vents also connect to Enterobacteria phages^13^. Similarly, there also found phage contigs identified as the *Microvirus* genus of Enterobacteria phages in methane seeps^43^. This suggests that similar bacterial families may be associated with the same phage families shared by different animal species or environments^13^.

### Gene expression of virus in *G. platifrons*

**Figure 4:**
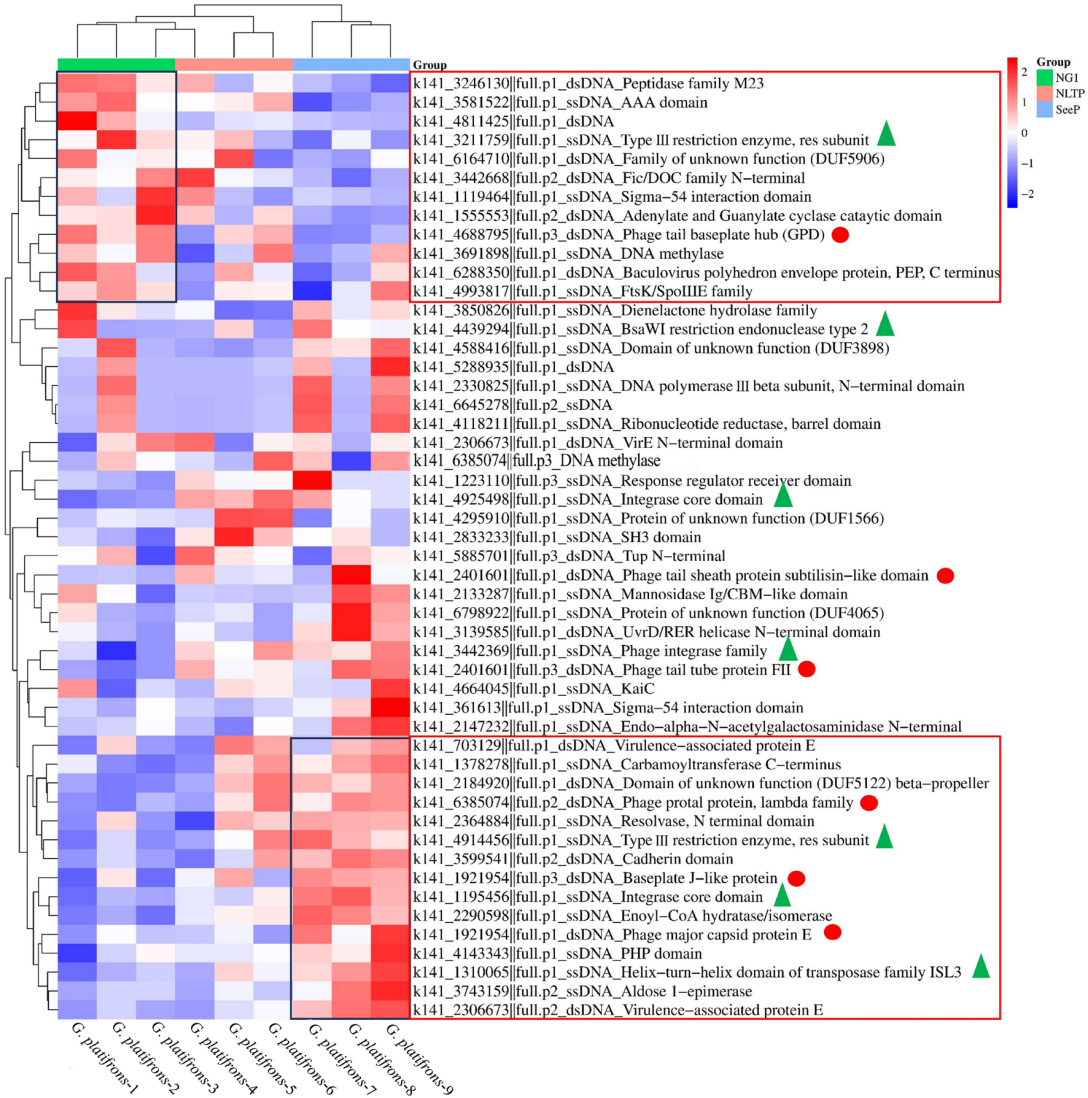
Expression analyses of viruses of *G. platifrons* in nine different individuals. mRNA data were obtained from the transcriptome of gill tissues for the generation of heatmaps. The color scale in the heatmap indicates expression values; blue color indicates low transcript abundance, and red color indicates high transcript abundance. The red circles represent viral structural genes, and green triangles represent integrase and restriction endonuclease genes. The highlighted squares represent highly expressed genes.

Transcripts from 50 genes of 47 different viruses, including both dsDNA and ssDNA types, were detected (Supplementary Table S4, Fig. 4). Among the predicted transcriptional genes were viral structural genes, integrase, restriction endonuclease, hydrolases, reductases, and other metabolically important enzymes. Interestingly, the integrase and restriction endonuclease genes were all from ssDNA, while most of the viral structural genes were from dsDNA (RNA phages were not considered). We analyzed the differential expression of viral transcriptomes in different environments; individuals from different environments showed significant variations in active viral expression (Fig. 4). In *G. platifrons*-1–3, which collected from an environment from low methane concentrations (up to 407 ppm), three genes involved in cell division were highly expressed: Fic/DOC family, FtsK/SpoIIIE family, and peptidase family M23 genes. In *G. platifrons*-7–9, which was collected from an environment with high methane concentrations (up to 4580 ppm), viral structural genes, integrase, and restriction endonuclease genes were highly expressed, indicating that the virus was more active in high methane concentrations. Additionally, aldose 1-epimerase, a key enzyme in carbohydrate metabolism, and enoyl-CoA hydratase/isomerase, an enzyme crucial in the fatty acid β-oxidative metabolic pathway, were highly expressed. Integrase and restriction endonuclease genes also exhibited high expression, suggesting that these two genes may serve as signature genes for virus integration into the bacterial genome. The results suggest the potential for gene exchange between viruses and bacteria in *G. platifrons*. Because of the nine individuals collected from different methane concentrations, the activity of viral composition had huge difference. It indicated that methane concentration is also critical for virus expression in *G. platifrons*.

### Virus–host interaction

**Figure 5:**
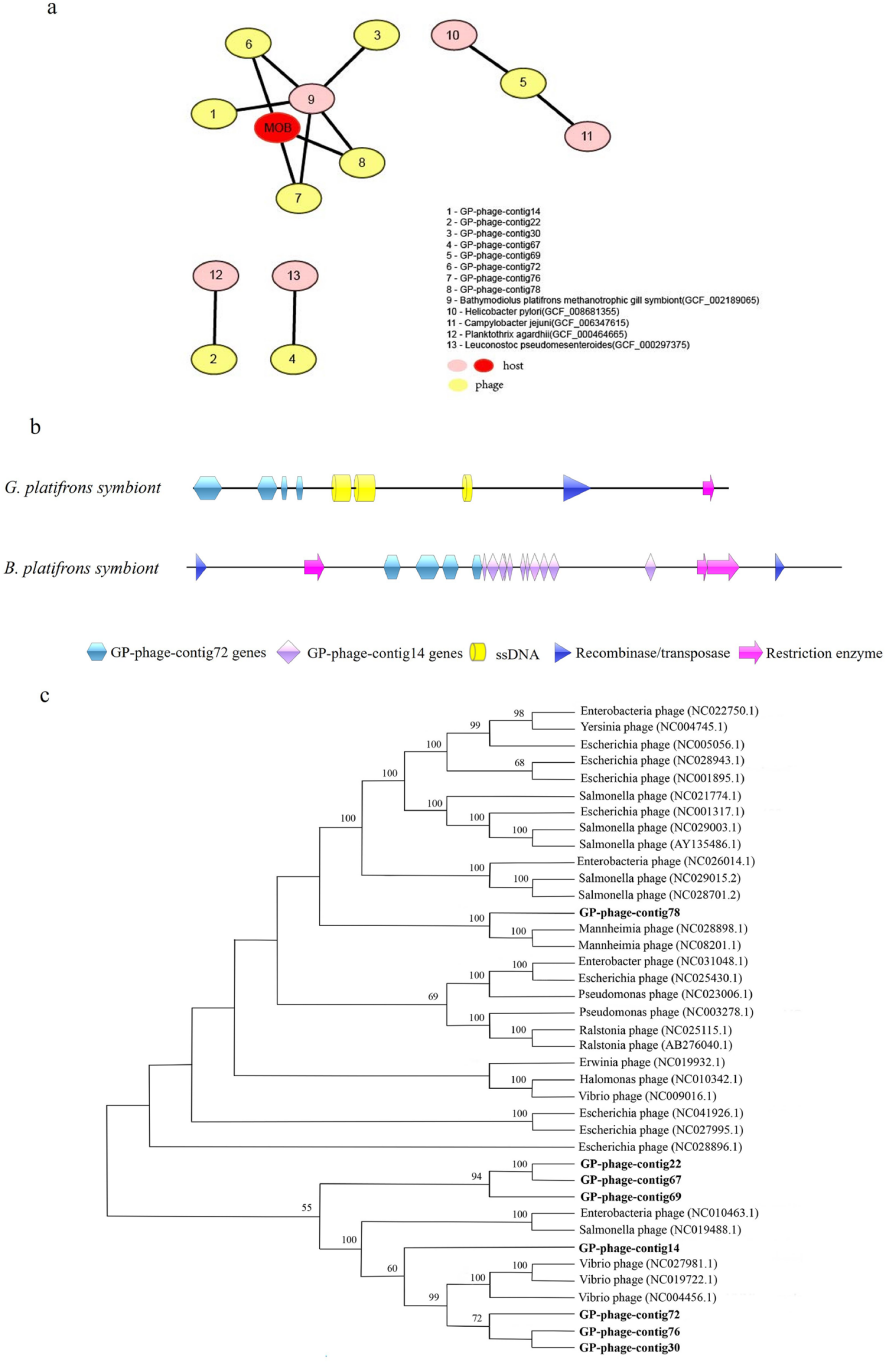
Predicted virus–host interaction. (**a**) Predicted virus–host network in *G. platifrons*. MOB is shown in red ovals, *G. platifrons* methanotrophic gill symbiont, which is a methane-oxidizing bacterium. The number in pink ovals represents other viral hosts listed beside. (**b**) Genome domains of the *G. platifrons* symbiont and the *B. platifrons* symbiont (GCF_002189065.1); (**c**) phylogenetic tree of viral contigs. There were 1000 bootstrap sub-replicates, and bootstrap values < 50 are not shown. The viral genome contigs of *G. platifrons* are shown in bold.

To investigate virus–host interactions in *G. platifrons* and understand the role of viruses in controlling host population dynamics and metabolic function, especially in relation to endosymbiotic bacteria, we employed CHERRY for species-level host prediction. We identified four interactions between viral contigs and prokaryotic genomes resulting from various types of interactions (e.g., protein organization information between viruses, sequence similarity between viruses and prokaryotes, and CRISPR signals). Four viral contigs (GP-phage-contig14, GP-phage-contig22, GP-phage-contig30, and GP-phage-contig67) were predicted to infect specific hosts, while four were linked to hosts from different prokaryotic bacteria. This result aligns with the common perception and previous findings that viral infections tend to be host-specific with a narrow host range^14,44,45^. The predicted hosts include *Methyloprofundus* sediment, *Bathymodiolus platifrons* methanotrophic gill symbiont, *Helicobacter pylori*, *Campylobacter jejuni*, *Planktothrix agardhii*, and *Leuconostoc pseudomesenteroides* (Fig. 5). Most viruses were linked to the methanotrophic symbiont in *G. platifrons*, which is a methane-oxidizing bacterium belonging to Gammaproteobacteria and plays a major role in the removal of the greenhouse gas methane from the biosphere. This result can be attributed to two main factors: the predominance of methanotrophic symbionts in the gill of *G. platifron*s and the similarity in virus community structure within the same tissue across different environments. GP-phage-contig14 and GP-phage-contig30, which specifically infected *B*. *platifrons* methanotrophic gill symbiont, indicate that virus–host interactions may be influenced by the environment.

In conjunction with the analysis of viral transcripts, we observed high expression of GP-phage-contig14 and GP-phage-contig72 (k141_1921954||full and k141_6385074||full) based on our data that found in *G. platifrons* and *B. platifrons* methanotrophic gill symbiont genomes (Fig. 5b) shown in Fig. 4. To investigate the interaction between the methanotrophic symbiont in *G. platifrons* and viruses, we annotated the genome sequences of *G. platifrons* and *B. platifrons* methanotrophic gill symbiont (GCF_002189065.1; Fig. 5b) and revealed the two virus (GP-phage-contig14 and GP-phage-contig72) activity in the two hosts. We found that viral genes had inserted into the host genomes, and these insertions were random and unstable. Additionally, the CRISPR/Cas and AbiEii domains (AbiE is a type IV toxin-antitoxin system, and AbiEii toxin is a putative nucleotidyltransferase containing a C-terminal domain) found adjacent to the insertion sites in the genome of *G. platifrons* methanotrophic gill symbiont, indicating the presence of multiple strategies in bacterial resist phage infection. In viral contigs that interacted with the host and exhibited transcript expression, the significant presence of ssDNA viruses was observed (*G. platifrons* methanotrophic gill symbiont contigs shown in Fig. 5b). Based on all the findings using the indirect bioinformatics methods, it is mere surmise that the integrase and restriction enzyme genes belonging to ssDNA may assist the insertion of viral genes (GP-phage-contig14 and GP-phage-contig72), which belong to dsDNA, into the host genome. As expected, more evidence is needed to confirm this surmise. Furthermore, there is substantial evidence indicating that interactions between viruses themselves are widespread^46–49^. When multiple viruses co-infect a single host, they can establish indirect interactions that alter host susceptibility, modify or suppress interferon response, or affect immune cell activation^50^, thereby avoiding superinfection^51^. A single virus infecting two or more hosts open the possibility of contact-based transfer of viral particles and/or genomes between or among syntrophic microbes, even those in different domains^52^. This could drive the rate of adaptive evolution of symbionts in different environments. The viral insertion element in the host genome serves two functions: assisting metabolism or defunctionalization.

Phylogenetic analysis was employed to investigate the homologous relationships among the viruses interacting with hosts, as shown in Fig. 5c. The results suggest that these viruses belong to the Gammaproteobacteria phage group. They can be clustered into two groups based on their host differences. Specifically, four viruses (GP-phage-contig14, GP-phage-contig30, GP-phage-contig72, and GP-phage-contig76) whose hosts were methane-oxidizing bacteria clustered with Vibrio and *Enterobacteria* phage, while one (GP-phage-contig78) clustered with *Mannheimia* phage belonging to the *Pasteurellales* phage. GP-phage-contig14, belonging to Myoviridae, showed a greater similarity to *Enterobacteria* phage during amino acid blast analysis with the NCBI database. This suggests that the host specificity is a distinctive feature of these viruses. However, the viral contigs were incomplete, preventing a comprehensive investigation of host functions. The identified function domains mainly involved infecting and regulating DNA transcription and replication. Previous studies have defined such incomplete viruses as defective interfering particles (DIPs)^53^. These particles exhibit critical absences in the viral genome, which can interfere with standard virus replication during coinfection^54^. However, it is important to note that we cannot conclusively confirm that the incomplete viral contigs in our study are indeed DIPs, as further evidence is required.

### Phylogenetic similarity between the virus and *G. platifrons* methanotrophic gill symbionts

**Figure 6:**
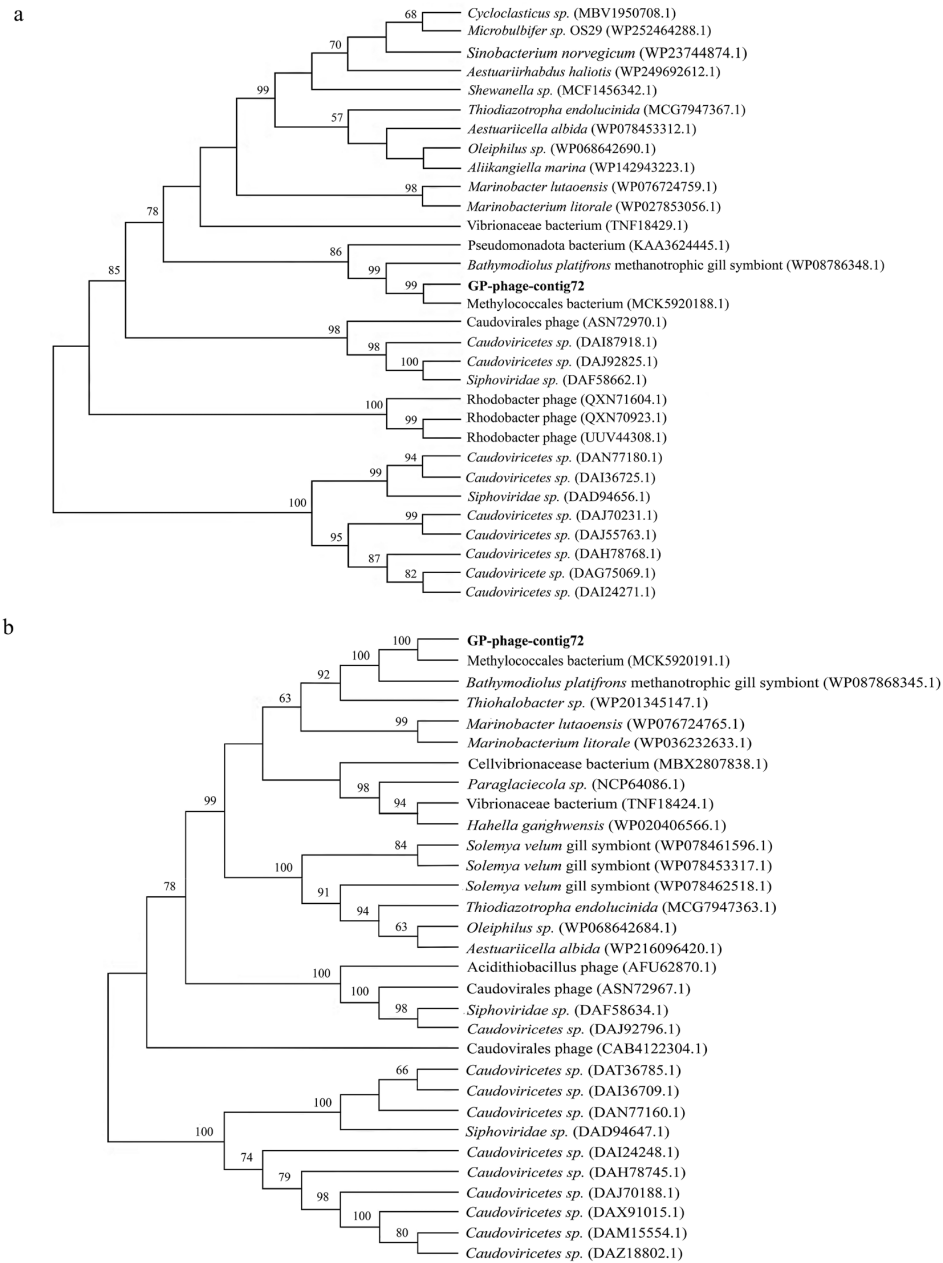
Phylogenetic analysis of Cro and DNA methylase genes of viruses and bacteria. (**a**) Phylogenetic tree of the Cro gene. (**b**) Phylogenetic tree of the DNA methylase gene. There were 1000 bootstrap sub-replicates, and bootstrap values < 50 are not shown. The viral contigs of *G. platifrons* are shown in bold.

Viruses selectively integrate certain bacterial genes when infecting phages (bacterial viruses) lyse the bacterial chromosome, a process akin to horizontal gene transfer^55^. These viruses carry virulence genes as an integral part of their genomes, and these transferred genes may be highly expressed when the viruses infect other bacterial hosts and initiate replication during a lytic cycle. In our study, both Cro and DNA methylase genes were found in viral contigs and bacterial genomes, indicating a potential horizontal gene transfer. To validate our hypothesis, a phylogenetic tree was constructed incorporating both viral and bacterial genes. The analysis revealed that the genes for Cro and DNA methylase had indeed phylogenetic similarity between GP-phage-contig72 and the *B. platifrons* methanotrophic gill symbiont (Fig. 6). Notably, these genes play a pivotal role in the lytic/lysogenic system. Transcriptomic analysis further demonstrated the expression of the DNA methylase gene in GP-phage-contig72 (Supplementary Table S2), affirming the utilization of the lytic/lysogenic system by the virus GP-Phage-contig72.

## Conclusions

In the deep-sea cold seep environment, viruses, bacteria, and *G. platifrons* must collaborate to adapt to the extreme conditions. We conducted an analysis of the viral metagenome and transcriptome extracted from *G. platifrons*, revealing a diverse viral community within the organism, including Baculoviridae, Rountreeviridae, Myoviridae, and Siphovirdae, but the active viromes were Myoviridae, Siphoviridae, and Podoviridae belonging to the Caudovirales order, with significant variations in abundance among individuals. The high expression levels of integrase, restriction endonuclease, and viral structural genes suggest an active virus lysis process. The activity of virus was affected by different methane concentrations. This led to an interesting surmise: ssDNA and dsDNA virus appeared to assist each other in infecting or lysing the host. Our findings provide additional evidence for the study of interactions between viruses and bacteria within *G. platifrons*, shedding light on the evolution of gene transfers between viruses and hosts. This serves as a foundational basis for further investigations into the role of microorganisms in *G. platifrons*.

## Methods

### Sample collection

*G. platifrons* specimens were collected from a cold seep in the South China Sea, located in the western Pacific Ocean (22°06.919′N and 119°17.140′E), at a depth of 1119 m, using the remotely operated vehicle Faxian aboard the research vessel Kexue in 2019. Gill tissues from nine individuals were stored at − 80 °C for subsequent DNA/RNA extraction. Of these nine mussels, three were collected from a seepage region with a high methane concentration, three were collected from a mussel bed with a moderate methane concentration, and the remaining three from regions with low methane levels. The mussels were promptly fixed with the RNAsafer stabilizer reagent (Omega Bio-Tek, Norcross, GA, USA) in situ and transferred to the deck within 1 h for use for extracting total DNA from the gill tissues.

### Metagenome sequencing and data from *G. platifrons* analysis

Total DNA was extracted from gill tissue samples of *G. platifrons* using the cetyl trimethylammonium bromide method^56^. The degree of DNA degradation and potential contamination was monitored on 1% agarose gels. DNA concentration was measured using the Qubit® dsDNA Assay Kit in a Qubit® 2.0 Fluorometer (Life Technologies, CA, USA). DNA samples with optical density values between 1.8 and 2.0 and a concentration above 1 µg were used to construct the library.

For DNA sample preparations, 1 µg of DNA per sample was used as input material. Sequencing libraries were generated using the NEBNext® Ultra DNA Library Prep Kit for Illumina (NEB, USA) following the manufacturer's recommendations. Index codes were added to attribute sequences to each sample. Briefly, the DNA sample was sonicated to a size of 350 bp, followed by end-polishing, A-tailing, and ligation with full-length adaptors for Illumina sequencing, followed by polymerase chain reaction (PCR) amplification. PCR products were then purified using the AMPure XP system, and libraries were analyzed for size distribution using an Agilent 2100 Bioanalyzer and quantified using real-time PCR.

The index-coded samples were clustered using a cBot Cluster Generation System according to the manufacturer’s instructions. After cluster generation, the library preparations were sequenced on an Illumina HiSeq platform, generating paired-end reads.

The original raw data from the nine individuals obtained from the Illumina HiSeq platform were cleaned using FastQC (v0.12.1) and Trimmomatic (v0.39) with custom parameters (ILLUMINACLTP: TruSeq3-PE.fa:2:30:10 SLIDINGWINDOW:4:15 LEADING:3 TRAILING:3 MINLEN:40 HEADCROP:12)^57^ for quality filtering (read length of 150 bp). Bowtie2 and samtools were then used to remove *G. platifrons* sequences^58,59^, and the *G. platifrons* genome was used to build the index database. The microbial sequence was obtained after removing the alignment of *G. platifrons* sequences. The trimmed reads of microorganisms in *G. platifrons* were assembled using Megahit with custom parameters (--k-min 21 --k-max 141 --k-step 10)^60,61^. The coverage of the assembled contigs was calculated with BBmap and Samtools^59,62^. The quality of the assembled contigs was evaluated using Quast^63^. Viral contigs were identified using VirSorter2 with default parameters, and the statistics of the virus sequences and viral type was performed using Excel (Fig. 2). The VirSorter2 database stores genetic information from viruses, which includes NCBI RefSeq, GenBank, PFAM, KEGG^64,65^, UniProt, EggNOG, and son on. The key criterion was the presence of viral hallmark genes (Table 2)^33^. The criteria for screening viral contigs were as follows: (1) viral genes > 0; (2) viral genes = 0 and (host gene = 0 or Virsorter2 viral hit max_score ≥ 0.95 or hallmark > 2); (3) manual check for those not falling under (1) and (2), with viral genes = 0 and host genes = 1 and length of ≥ 10 kb; (4) discarding the rest^66^. CheckV was used to evaluate the quality of viral metagenome contigs, including the identification of host contamination of proviruses, estimation of genomic fragment integrity, and identification of closed viral genomes^65^. Prodigal (version 2.6.3) was used to predict the coding sequences (CDS) of the viral genomes extracted from VirSorter2^31^. Predicted CDS were then annotated using HMMER3 (version 3.3) against a custom comprehensive viral HMM database, which includes Xfams (as described in the “Methods” Section) and viral protein families from the JGI Earth’s virome project^67,68^.

**Table 2:** Hallmark genes of different virus types.

Virus type	Hallmark genes
dsDNA phage	baseplate, coat, fiber, head, major-capsid-protein, portal, spike, structure, tail, terminase-large-subunit, virion-formation
ssDNA	phage_F, viral_Rep
RNA	rdrp
NCLDV	MCP, pATPase, primase, TFIIs, VLTF3
Lavidaviridae	MCP

### RNA-Seq and data of virus of the transcriptome from *G. platifrons* analyses

Transcriptome sequencing was performed using Novogene (Tianjin, China) as described previously. Briefly, gill tissues from mussels were subjected to RNA extraction using TRIzol reagent (Invitrogen, MA, USA). RNA quantification, mRNA purification, cDNA synthesis, adaptor ligation, cDNA library construction, and sequencing using the Illumina HiSeq 2500 platform with paired-end reads were conducted using Novogene (Tianjin, China).

Abundance of viral transcriptome reads was computed using Bracken, which assigns taxonomy labels using Kraken 2 software with default parameters to estimate the number of viral reads present in each sample^69,70^. The viral taxonomy was based on alignment against the NCBI RefSeq database.

The trimmed reads of the *G. platifrons* transcriptome were obtained through a quality check using FastQC, followed by trimming using Trimmomatic. The *G. platifrons* sequences were then removed using Bowtie and samtools. The resulting trimmed reads were assembled de novo into a transcript using the paired-end method with Trinity^71^. Trinity consists of three software modules: Inchworm, Chrysalis, and Butterfly. In the first step in Trinity, the Inchworm module assembles the RNA-seq data into longer, gapless unique sequences called contigs. Next, chrysalis clusters the Inchworm contigs, representing the full transcriptional complexity for a given gene (or sets of genes that share common sequences). Chrysalis then partitions the full-read set among these clusters. Finally, Butterfly processes the individual graphs in parallel, tracing the paths that reads and pairs of reads take within the graph. It ultimately reports full-length transcripts for alternatively spliced isoforms and separates transcripts corresponding to paralogous genes. The resulting sequences are referred to as unigenes.

Abundance estimation was calculated using the expectation–maximization method^72^. The trimmed mean of M values method was employed to normalize the abundance^73^. Differentially expressed unigenes in the samples of *G. platifrons* virus from areas with different methane concentrations were identified using edgeR with a log2 fold change (Log2FC) > 0.5 (upregulated) or Log2FC <  − 0.5 (downregulated) and false discovery rate (FDR) < 0.05^74^.

### Phage protein clustering and taxonomic comparisons

Diamond was used to map the predicted ORFs against the NCBI Viral RefSeq database^75^. The Markov clustering algorithm (MCL) was used to group viral protein clusters (PCs), and ClusterONE was used to format the VCs^76,77^. The similarities between viral genomes were calculated using a one-tailed P value^37^. These steps were all conducted by vConTACT2 with default parameters (--rel-mode ‘Diamond’ --proteins-fp test_data/proteins.csv --db 'ProkaryoticViralRefSeq94-Merged' --pcs-mode MCL --vcs-mode ClusterONE)^78^. The viral protein network formed from vConTACT2 was visualized with Cytoscape software (v.3.6.0; http://cytoscape.org/), where nodes represent genomes and edges connect significantly similar viral genomes^37,79^.

### Host prediction of viral contigs

Virus–host connections were predicted using the CHERRY method, considering various prediction types including protein organization, CRISPR, sequence similarity, and k-mer frequency^79^. MCL was used to construct PC, with reference viral genome proteins downloaded from NCBI RefSeq. Prodigal was used for gene finding and protein translation of query contigs^67^. Diamond was used to blastp the viral proteins against NCBI RefSeq with an e-value of 1e−5, followed by clustering proteins with an inflation value of 2.0 through MCL^76^. Prediction on the species level used pretrained parameters (--len 5000 –model pretrain –topk 1). The virus–host connection network was prediction in CHERRY^78^ also visualized in Cytoscape software (v.3.6.0; http://cytoscape.org/), with nodes representing host genomes and edges connecting significantly similar viral genomes^80^. The viral contigs and the *G. platifrons* methanotrophic gill symbiont genome annotated using TrinotateFFAM and KEGG database and visualized using IBS 1.0.3 software (Fig. 5b).

### Phylogenetic analysis

To study the homologous relationship between viruses interacting with hosts, a neighbor-joining tree was created for viruses and bacteria contigs from the NCBI database using MEGA v11^81^. Bootstrap sub-replicates were set to 1000, and bootstrap values less than 50 were not obtained.

### Supplementary Information

Supplementary Information 1.

Supplementary Information 2.

Supplementary Information 3.

Supplementary Information 4.

## Data Availability

All data needed to evaluate the conclusions in the paper are presented in the paper and/or the supplementary materials. All metagenome and transcriptome sequencing data have been deposited with links to BioProject accession number PRJNA1040436 in the NCBI BioProject database (https://www.ncbi.nlm.nih.gov/bioproject/).
